# To Believe Is Not to Think: A Cross-Cultural Finding

**DOI:** 10.1162/opmi_a_00044

**Published:** 2021-09-10

**Authors:** Neil Van Leeuwen, Kara Weisman, Tanya Marie Luhrmann

**Affiliations:** Department of Philosophy and Neuroscience Institute, Georgia State University; Department of Psychology, University of California, Riverside; Department of Anthropology, Stanford University

**Keywords:** belief, thinking, credence, cognitive attitudes, epistemic verbs, religious psychology, theory of mind

## Abstract

Are religious beliefs psychologically different from matter-of-fact beliefs? Many scholars say no: that religious people, in a matter-of-fact way, simply think their deities exist. Others say yes: that religious beliefs are more compartmentalized, less certain, and less responsive to evidence. Little research to date has explored whether lay people themselves recognize such a difference. We addressed this question in a series of sentence completion tasks, conducted in five settings that differed both in religious traditions and in language: the United States, Ghana, Thailand, China, and Vanuatu. Participants everywhere routinely used different verbs to describe religious versus matter-of-fact beliefs, and they did so even when the ascribed belief contents were held constant and only the surrounding context varied. These findings support the view that people from diverse cultures and language communities recognize a difference in attitude type between religious belief and everyday matter-of-fact belief.

## INTRODUCTION: VARIETIES OF BELIEF

Many people have beliefs about gods, ancestors, souls, and other supernatural phenomena. Indeed, religious beliefs have shaped human history for millennia. But people also have beliefs about whether it will rain, where their children are, or what time of year it is; such matter-of-fact beliefs are central to everyday life.

Do people’s religious beliefs typically involve the same cognitive attitude as their matter-of-fact beliefs? The phrase “cognitive attitudes” refers to the various ways humans process and relate to ideas about the world (Shah & Velleman, 2005; Van Leeuwen, 2014). A person might *think* that it’s about to rain, *wonder* whether it’s about to rain, *assume*, *doubt*, *imagine*, *pretend*, *hope*, or *worry* that it’s about to rain. At stake here is whether religious ideas—for all their importance—are usually processed in the same way as the matter-of-fact belief that it is about to rain.

Many scholars say the cognitive attitudes are the same (Boudry & Coyne, 2016a, 2016b; S. Harris et al., 2009; Levy, 2017; Talmont-Kaminski, 2016). On this view, religious people just think gods and spirits exist, just like anyone simply thinks tables and chairs exist. Others hold that religious beliefs are different: more compartmentalized (Astuti & Harris, 2008; P. L. Harris & Giménez, 2005; Watson-Jones et al., 2016), more effortful (Boyer, 2013; Luhrmann, 2018), in need of regular voluntary practice (Luhrmann, 2012; Norenzayan, 2013), less certain (Clegg et al., 2019; Davoodi et al., 2018; P. L. Harris et al., 2006), less responsive to counterevidence (Liquin et al., 2020; Stanovich & Toplak, 2019; Van Leeuwen, 2017), and experienced as part of one’s identity (Durkheim, 1912/2008).

This debate concerns not the *contents* of religious vs. matter-of-fact belief but the *attitude*, or the *way* people relate to contents or ideas. Here is a philosophical characterization of this difference: For any content *p* (supernatural or naturalistic; scientific or not; observable or unobservable, etc.), one can relate to that content in various ways. Consider the following attitude reports:

1)  Karen doubts *that it will rain*.
2)  Sarah hopes *that it will rain*.
3)  Bob doubts *that God exists*.
4)  Arthur hopes *that God exists*.

The italicized attitude contents reported in 1 and 2 are naturalistic, while 3 and 4 concern the supernatural. But the attitudes of doubting and hoping can occur with either kind of content. Now consider the attitudes one might hold toward these contents:

5)  Jane factually believes *that Jesus was born in Bethlehem*.
6)  Fred has the religious belief *that Jesus was born in Bethlehem*.

Despite having the same contents, one might suggest that the second mental state, 6, is a reverential, identity-constituting attitude that is compartmentalized, less responsive to evidence, and less widely used than factual belief to support ordinary inferences. One of us (Van Leeuwen, 2014), for example, has argued that this is a much more typical attitude for religious contents. Others, however, such as Levy (2017), deny that there is such a difference in “belief” type: Religious beliefs are just factual beliefs that happen to be about supernatural contents (hence the title of his paper: “Religious Beliefs Are Factual Beliefs”). So the dispute comes down to whether a meaningfully different cognitive attitude of religious credence exists at all.

In this article, we present new data that speak to this important debate. Our studies tested whether lay people themselves recognize a difference between matter-of-fact and religious belief. We predicted that people from diverse cultural and religious settings would systematically choose different words for describing matter-of-fact vs. religious cognitive attitudes, thereby revealing that they distinguish them.

To date, one series of studies has addressed such a prediction. Heiphetz et al. (2021) used corpus analyses as well as sentence completion tasks to show that American English speakers are more likely to use the word “believe” to describe religious beliefs (e.g., “Zane believes that Jesus turned water into wine”), but “think” to describe matter-of-fact beliefs (e.g., “Nick thinks that George Washington was the first U.S. president”). Importantly, this was so even when they held reported attitude contents constant and varied the situation to make the surrounding context religious or nonreligious. They thus concluded not only that there are distinct attitudes, but also that lay people are at some level aware of the difference and use available resources to express it.

These earlier studies, however, left an important question open: How widespread is this distinction across cultures? Many observers hold that “religious belief” is only a Western, Christian, and perhaps modern idea: Other people do not believe—they know (Asad, 1993; Smith, 1977). In a representative statement, Toren (2007, p. 307) writes: “We [anthropologists] may characterise as *belief* what our informants *know* and, in so doing, misrepresent them.” The implication is that the distinction surfaced in Heiphetz et al. (2021) does not appear in non-Western, non-Christian contexts. Some anthropologists, however, have found that religious belief is indeed a distinct attitude in various non-Western cultures—for example, the Vezo in Madagascar and the Fang in Central Africa (Astuti & Harris, 2008; Boyer, 2013).

Following recent calls to assess psychological theories in a variety of cultural settings (Henrich et al., 2010), we set out to test empirically whether people differentiate matter-of-fact and religious belief in culturally diverse fieldsites. The studies we present here were part of a larger project investigating cultural models of the mind and their relationship to spiritual experiences, which took place in five countries—from west to east: the United States, Ghana, Thailand, China, and Vanuatu—chosen to include a variety of spiritual and religious practices (for more details on the project, see Luhrmann, 2020; Luhrmann et al., 2021). Participants were primarily Christian or religiously unaffiliated in the United States, overwhelmingly Christian in Ghana and Vanuatu, Buddhist in Thailand, and religiously unaffiliated in China.

For purposes of designing our study stimuli, we used a “minimum definition” of religion that would be useful cross-culturally: thought, talk, and practices that concern gods, spirits, or other supernatural beings (cf. Goody, 1961). Even in religious traditions that *seem* to have no spirits (such as Buddhism), participants often have ideas and practices about a wide range of spirits (Gross, 2002). That said, we used this definition provisionally, and we hold open the possibility that in some cultural contexts people’s religious attitudes can concern other subject matters as well (see General Discussion).

In the United States, studies were conducted in English and featured the words *think* and *believe*, following Heiphetz et al. (2021). For other countries, counterparts to “think” and “believe” were chosen in consultation with native speakers and anthropologists with local expertise. These counterparts were not expected to be exact semantic matches. Rather, we predicted that people would use them in parallel ways to track the distinction between religious and matter-of-fact belief. In Ghana, studies were conducted in Fante (an Akan dialect) and focused on the words *dwen* and *gye dzi*. In Thailand, studies were conducted in Thai and focused on the words คิด (*kit*) and เชื่อ (*chưa*). In China, studies were conducted in Mandarin (in the variety known as Standard Chinese) and focused on the words 认为 (*rènwéi*) and 相信 (*xiangxìn*). In Vanuatu, studies were conducted in Bislama (an English-based creole) and focused on the words *ting* and *bilif*. All study materials were back-translated to ensure accuracy.

To be clear: Our main interest in these studies was not in the semantics of these pairs of words per se, but in the way that people use them to report distinct cognitive attitudes. Our hypothesis was that the ability to do so is widespread across cultures; we predicted that people would think differently about religious and matter-of-fact beliefs and that these different understandings would manifest as differential use of words like “think” and “believe” in sentence completion tasks. We have not in these studies addressed the following questions: (1) How many *other* ways can the words we focused on be used, aside from the ways we focus on here? (2) To what extent do the verb pairs we focus on have resembling semantic profiles across languages, aside from being close enough to facilitate a test of our prediction? (3) How did cultural influences shape the semantics and pragmatics of these words over historical time? Beyond providing a test for the hypothesis we have fleshed out here, we hope that the studies that follow provide a launching point for investigating those related questions.

Our three studies, which closely follow Heiphetz et al.’s (2021) experimental studies, each tested whether lay people use the pairs of words indicated here to communicate a difference between matter-of-fact and religious beliefs. For each study, we predicted that participants would be more likely to use “believe” (or its hypothesized counterpart) for religious belief than for matter-of-fact belief. (Our studies were preregistered at https://aspredicted.org/p6iy3.pdf; see the Supplemental Materials for complete preregistered analyses, as well as further details on method.)

## STUDY 1: FORCED CHOICE (“THINK” VS. “BELIEVE”)

Study 1 provided an initial test of our prediction.

Participants (*N* = 344; *n* = 48–97 per site) were presented with 25 sentences in one of two counterbalanced orders. Each sentence had the form “[Character] [thinks / believes] that X,” where X was a propositional phrase (e.g., *John [thinks / believes] that Jesus Christ died for human sins*). Participants selected one of the two words to complete the sentence. In the United States, Thailand, China, and Vanuatu, participants completed a pen-and-paper survey. In Ghana, the study was administered orally at a rural site, since Fante is rarely written; at this location, a research assistant read each item out loud and recorded verbal responses.

Ten of these sentences were “religious”: The complement phrase included Christian content (e.g., *Jesus Christ died for human sins*) or Buddhist content (e.g., *the Buddha found spiritual truth while meditating*).

The remaining 15 sentences were “matter-of-fact”: The complement included a widely known fact (e.g., *Brazil is in South America*), a less widely known fact (e.g., *bronze contains more copper than tin*), or a personal life fact (e.g., *her dad is cooking noodles for dinner*).

**Figure 1.**
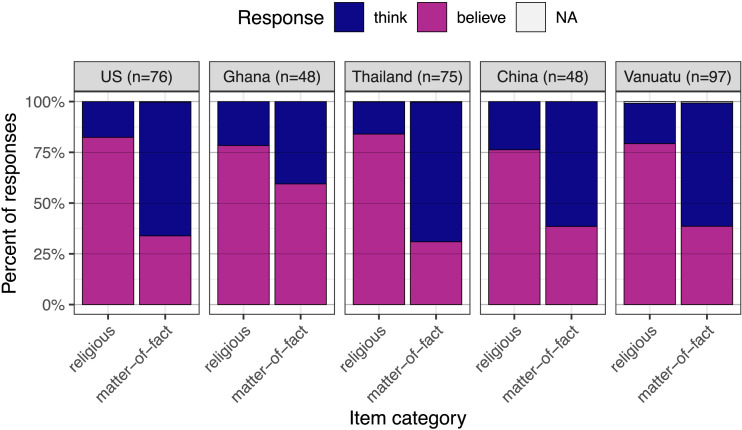
**Study 1 results.** Participants in all fieldsites were more likely to select “believe” (or its counterparts in other languages) to complete religious vs. matter-of-fact sentences.

A mixed effects logistic regression revealed that, as predicted, participants were generally more likely to select “believe” (or its counterpart) to complete religious vs. matter-of-fact attitude ascriptions (β = .20, *p* < .001). This distinction was more pronounced in Thailand and the United States, and less pronounced in Ghana; it did not differ from the grand mean in China or Vanuatu (see Table S3 for complete results). Nonetheless, secondary analyses confirmed that this difference was significant in each fieldsite considered alone (United States: β = .24, *p* < .001; Ghana: β = .09, *p* = .010; Thailand: β = .27, *p* < .001; China: β = .19, *p* < .001; Vanuatu: β = .20, *p* < .001; see Table S4 and Figure 1).

## STUDY 2: FREE RESPONSE

In Study 2, a separate group of participants (*N* = 388; *n* = 46–100 per site) completed the same sentences using a word or phrase of their own free choice. Methods were otherwise identical to Study 1.

**Figure 2.**
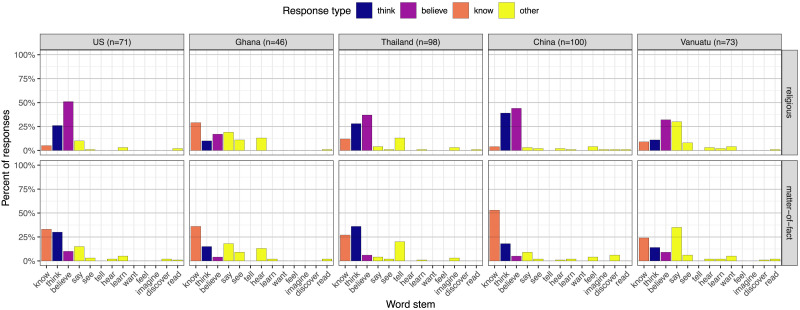
**Study 2 results.** Participants in all fieldsites were more likely to generate responses containing the word stem “believe” (or its counterparts) to complete religious vs. matter-of-fact sentences. This plot presents 13 word stems that include the six most common responses in each fieldsite, ordered by prevalence.

Echoing Study 1, participants were more likely to generate responses containing the word-stem “believe” (or its counterpart) for religious compared to matter-of-fact attitude ascriptions (β = .13, *p* < .001). Indeed, in four of these five sites, “believe” (or its counterpart) appeared in the plurality of free responses to religious sentences; see Figure 2. The difference between religious and matter-of-fact sentences was more pronounced in the United States and China, less pronounced in Ghana and Vanuatu, and did not vary from the grand mean among participants in Thailand (see Table S11). But again, this difference was significant in each fieldsite considered alone (United States: β = .20, *p* < .001; Ghana: β = .06, *p* < .001; Thailand: β = .14, *p* < .001; China: β = .17, *p* < .001; Vanuatu: β = .09, *p* < .001; see Table S12).

## STUDY 3: VIGNETTE COMPLETION

Study 3 (*N* = 328; *n* = 49–80 per site) provided a final, closely controlled test of our primary claim. Methods were similar to Study 1, except that, rather than completing attitude ascriptions that were either religious or matter-of-fact in content, there were five pairs of brief vignettes that set up either a matter-of-fact or religious context, but ended with the same final sentence that the participant had to complete. For example:

Kerry had bad headaches in the afternoons all last year. Sometimes her friends offered her aspirin. But Kerry took courses at a medical school. That school teaches that drinking water is the way to cure a headache and aspirin is not. So Kerry always refused the aspirin her friends offered. That’s because she [ thinks / believes ] that aspirin is not a cure.

Terry had bad headaches in the afternoons all last year. Sometimes her friends offered her aspirin. But Terry belonged to the Church of Christ Scientist. That church teaches that prayer is the way to cure illness and medicine is not. So Terry always refused the aspirin her friends offered. That’s because she [ thinks / believes ] that aspirin is not a cure.

This allowed us to test ascriptions of cognitive attitudes while holding the ascribed content constant. In this case, Kerry and Terry might be ascribed different attitudes to the same content: *that aspirin is not a cure*.

Vignettes were presented in one of two counterbalanced orders, with paired vignettes separated from each other. “Religious” vignettes included diverse religious traditions, and “matter-of-fact” vignettes were scientific, historical, or commonsensical in nature.

**Figure 3.**
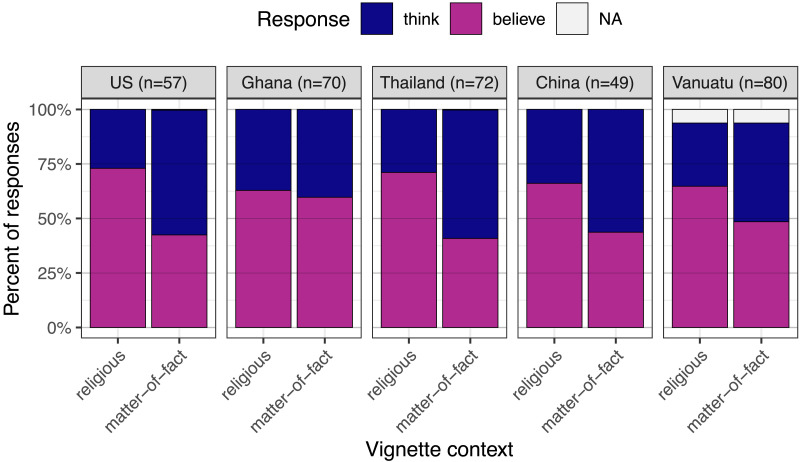
**Study 3 results.** Participants in the United States, Thailand, China, and Vanuatu—but not Ghana—were more likely to select “believe” (or counterparts) to complete an attitude report when it was embedded in a religious (vs. matter-of-fact) vignette.

As predicted, participants were generally more likely to select “believe” (or its counterpart) when the sentence was embedded in a religious vignette as opposed to the matching matter-of-fact vignette (β = .10, *p* = .010). This distinction was more pronounced in the United States and Thailand, less pronounced in Ghana, and did not differ from the grand mean among participants in China or Vanuatu (see Table S20). The difference was significant in four of the five sites considered alone (United States: β = .15, *p* < .001; Thailand: β = .15, *p* = .009; China: β = .11, *p* = .033; Vanuatu: β = .08, *p* = .049; see Table S21 and Figure 3). The difference was not significant in our sample from rural Ghana (β = .02, *p* = .747), but it did appear to be present in a sample of Ghanaian undergraduates in a small follow-up study (see the Supplemental Materials).

## GENERAL DISCUSSION

To step back, across Studies 1 and 2, participants were generally more likely to use the word “believe” (or its counterparts in other languages) to describe cognitive attitudes that relate to gods, ancestors, souls, and other supernatural phenomena, than to describe matter-of-fact beliefs. In Study 3, which featured cognitive attitudes with neutral contents, participants were more likely to use “believe” when the surrounding vignette made clear that the attitude in question was held in a religious context than when the surrounding vignette provided a matter-of-fact context.

We consider this to be evidence that matter-of-fact beliefs and religious beliefs involve distinct cognitive attitudes, that awareness of the difference is widespread among human cultural groups, and that this distinction manifests in the word choices of people speaking a wide variety of languages.

Across all our studies and sites, there was only one exception to the pattern that held everywhere else: Among Ghanaian participants, preferential use of *gye dzi* (the Fante counterpart to “believe”) for religious as opposed to matter-of-fact attitudes was present, but attenuated, in Studies 1–2, and absent in Study 3. This may simply be an experimental artifact, but it raises the intriguing possibility that in Ghana—a setting where thought and talk about the supernatural is quite commonplace (Dulin, 2020; Dzokoto, 2020)—many people hold more matter-of-fact attitudes about religious ideas (see the Supplemental Materials for further discussion). Conversely, there may also be cultural settings or spheres of discourse in which people hold “religious” belief attitudes about factual content; for example, recent years have made it clear that beliefs about such “here-and-now” matters as disease transmission and election security can become central parts of people’s identities and can be extremely difficult to counter with factual evidence.

Our studies ruled out possible alternate explanations for the main pattern we found (discussed in more detail in the Supplemental Materials).

First, if participants used the word “believe” (or counterparts) *merely* to indicate that the protagonist of the sentence or vignette was uncertain or unconfident, then we would likely have seen greater incidence of “believe” for less well-known content—items designated a priori as “less-widely-known facts”—than for well-known facts in Studies 1–2 (for a discussion of the relation between belief confidence and perceived consensus, see Shtulman, 2013, p. 207). But no such pattern emerged, so mere ascription of lesser confidence is unlikely to be what participants were indicating with their differential word choice.

Second, if participants used the word “believe” only to indicate that they themselves disagreed with the attributed attitude, then we likely would have found an effect of religion, with people using “believe” (or counterparts) more for religions other than their own. No such pattern emerged. In fact, particularly in the most devoutly Christian samples—in Ghana and Vanuatu—people were even more likely to use “believe” in sentences with content from their own religion.

Third, if “believe” reflected only religious *content* (rather than a difference in cognitive attitude), then we wouldn’t have seen the differences that we saw in Study 3, in which propositional complements were matched exactly and only the broader context varied.

In fact, Study 3 also addressed a range of further content-based explanations of the effects that surfaced in Studies 1 and 2. One might, for example, argue that the effects in Studies 1 and 2 emerged because people were using “believe” for unobservable subjects and “think” for observable subjects, or that they were using “believe” for ideas that were matters of opinion and “think” for objective matters. But for Study 3, these distinctions can’t be the explanation: with attitude contents held fixed (e.g., *that aspirin is not a cure*), there could not be a difference in what the respective attitudes were about.

Nonetheless, these studies raise a range of interesting questions for future research. Might differential use of verbs like “believe” vs. “think” also indicate differences between moral and nonmoral cognitive attitudes, between beliefs arrived at reflectively vs. intuitively, or between more and less speculative cognitive attitudes in science? Finding such patterns would not conflict with the results here; rather, they would complement our overall outlook by affirming that people in general have the cognitive flexibility to understand and express nuanced differences in cognitive attitude type.

Fourth and finally, if the differentiation between matter-of-fact belief and religious belief were unique to Western contexts, we would not have seen the striking similarities across cultures that we did.

Our results are most parsimoniously explained by our main hypothesis: Matter-of-fact belief and religious belief are distinct cognitive attitudes, and people in many different cultures and language communities are aware of the difference. The cognitive flexibility needed to utilize and differentiate these attitudes is not specific to Westerners, Christians, scholars, or some other rarefied group; instead, it appears to be widely shared. Matter-of-fact beliefs are likely used in a problem-solving way to achieve practical goals, while religious beliefs are used in guiding symbolic actions expressive of sacred values (Atran & Axelrod, 2008); thus, tracking the distinction between them may allow people to better understand and predict others’ behaviors. Indeed, this distinction may be one of the common features of a theory of mind that we increasingly understand to be subtle and sophisticated across social worlds.

## ACKNOWLEDGMENTS

Thanks especially to the team leaders at each fieldsite: Joshua Brahinsky and Nikki Ross-Zehnder (United States), John Dulin and Vivian Dzokoto (Ghana), Felicity Aulino (Thailand), Emily Ng (China), and Rachel Smith (Vanuatu). Thanks to Dan Weiskopf for insight into the hypotheses tested here, to Larisa Heiphetz for help with study design and preregistration, and to Jonathan Jong, Kilu von Prince, and Rebecca Tuvel for feedback on earlier drafts. We also thank the editors of *Open Mind* and three anonymous referees for feedback. See the Supplemental Materials for further acknowledgments.

## FUNDING INFORMATION

TL, John Templeton Foundation (https://dx.doi.org/10.13039/100000925), Award ID: 55427. KW, National Science Foundation (https://dx.doi.org/10.13039/100000001), Award ID: DGE-114747.

## AUTHOR CONTRIBUTIONS

NVL: Conceptualization: Lead; Investigation: Equal; Methodology: Equal; Writing – Original Draft: Lead; Writing – Review & Editing: Equal. KW: Data Curation: Lead; Formal Analysis: Lead; Validation: Lead; Visualization: Lead; Writing – Original Draft: Supporting; Writing – Review & Editing: Equal. TL: Conceptualization: Supporting; Investigation: Equal; Methodology: Equal; Project Administration: Lead; Supervision: Lead; Writing – Original Draft: Supporting; Writing – Review & Editing: Equal.

## Supplemental Materials

https://osf.io/qy3js/

https://github.com/kgweisman/think_believe
