# Supplementary material for: To Believe Is Not to Think: A Cross-Cultural Finding
Source: Open Mind (Camb). 2021 Sep 10;5:91–9. doi: 10.1162/opmi_a_00044 (PMC8563061; doi:10.1162/opmi_a_00044)
Supplement: Supplementary file 1 [file opmi-05-91-s001.pdf]

Supplementary Materials for

To Believe is Not to Think: A Cross-Cultural Finding

Neil Van Leeuwen, Kara Weisman, Tanya Marie Luhrmann.

Correspondence to Neil Van Leeuwen: nvan@gsu.edu

This file includes:

Materials and Methods
Supplementary Text
Figs. S1 to S3
Tables S1 to S24
Captions for Data S1 to S3

Other Supplementary Materials for this manuscript include the following:

Data S1 to S3

Materials and Methods

In three studies, we tested whether lay people use the English words "think" and "believe" (or counterparts in four other languages) to communicate a distinction between matter-of-fact and religious beliefs. For each study, we predicted that participants would be more likely to use "believe" (or its counterpart) for religious belief than for matter-of-fact belief. These studies closely followed Heiphetz, Landers, and Van Leeuwen's (2021) experimental studies (Studies 2-4). All studies were preregistered at https://aspredicted.org/p6iy3.pdf; here we provide both the preregistered analyses and the more sophisticated repeated measures analyses highlighted in the main text.

Samples

The current studies were part of the Mind and Spirit project, a Templeton funded, Stanford-based comparative and interdisciplinary project under the direction of T.M. Luhrmann (PI), drawing on the expertise of anthropologists, psychologists, historians, and philosophers.The project asked whether different understandings of "mind," broadly construed, might shape or be related to the ways that people attend to and interpret experiences they deem spiritual or supernatural. This project utilized a mixed method, multiphase approach, combining participant observation, long form semi-structured interviews, quantitative surveys among the general population and local undergraduates, and psychological experiments with children and adults. We worked in five different countries: the US, Ghana, Thailand, China, and Vanuatu. We chose these sites because they offer a range of cultural models of the mind and a variety of religious and spiritual traditions (Luhrmann, 2020; Luhrmann, Weisman, et al., 2021).

The studies reported here primarily sampled urban university students in the San Francisco Bay Area, US; Chiang Mai City, Thailand; Shanghai, China; and Port Vila, Vanuatu (as well as one sample of urban university students in Cape Coast, Ghana, to extend our findings in Study 3; see "Study 3b, Ghanaian undergraduates (English)," below). When working with university students, local research assistants recruited participants on university campuses and handed them pen and paper surveys to fill out. In the US, studies were conducted in English. In Thailand, studies were conducted in Thai. In China, studies were conducted in Mandarin (Standard Chinese variety), written in simplified script. In Vanuatu, studies were conducted in Bislama (an English-based creole).

We also included samples of Ghanaian Fante-speakers to diversify the range of languages spoken by our participants. Because the majority of Fante-speakers in university settings in Ghana also speak English (the language of instruction), a highly skilled research assistant recruited Fante-speaking samples in the rural villages surrounding Cape Coast; these participants were not undergraduates, and did not speak English as their primary language. Because Fante is not commonly a written language, these participants were interviewed face-to-face and their oral responses were recorded by the research assistant.

Both within and across sites, participants had varying degrees of exposure to English. However, in Thailand and Ghana the majority of participants did not speak English, and in no site except for the US was English a first language for most participants. See Table S1 for demographic information about these samples, including English exposure.

Study 1

Participants. The final sample for analysis included 344 adults (US: n=76; Ghana: n=48; Thailand: n=75; China: n=48; Vanuatu: n=97). See Table S1 for demographic information. We

aimed to recruit at approximately 50 participants per site, but retained any participants that exceeded this minimum sample size. An additional 33 participants were excluded from analyses because they completed this task after completing other experimental surveys (n=29), or because they failed an attention check (see "Procedure"; n=4).

Procedure. For all participants included in our final sample, this task was the first task included in a larger packet of survey measures. Instructions for the US participants read, "In this survey, you will read twenty-five sentences with a missing word. You should complete each sentence by *circling* a version of either the word 'think' or the word 'believe.' Please take your time to make sure that the word you choose makes the most sense in the sentence as a whole. *Pick only one for each sentence*. If both words seem acceptable, pick the one that seems to you to fit better. It is important that the sentence should be grammatical. After you have circled an answer, do not go back and change it. After the survey items, there will be one question to check attention and a few questions about yourself. When you are done with the survey, please return it to the research assistant who gave it to you." Instructions for participants at other locations were literal translations of this passage in the relevant language. In all locations except for Ghana, participants read these instructions before completing the survey with pen and paper; in Ghana, a research assistant read the instructions (slightly modified for oral interaction) aloud to participants, before administering the task orally.

The task consisted of 25 sentences in the relevant local language presented in one of two orders (counterbalanced across participants within each sample). Ten of these sentences were "religious": The complement phrase included either Christian or Buddhist content. The remaining 15 sentences were "matter-of-fact": The complement included either a widely-known fact, a less-widely-known fact, or a personal life fact. See Table S2 for the full list of items in English; Study 1 items in other languages were translated and backtranslated to ensure accuracy.

Participants then completed an attention check, located on a separate page after the 25 forced choice questions. Instructions in English read, "On the previous pages, you filled in words to describe what people think or believe. Without turning back, please write below what one of the sentences was about. You do not need to remember the item word-for-word; just the general idea is fine." Instructions in other languages were a literal translation of this. The data entry team determined whether participant passed or failed the attention check. Participants passed if they wrote at least approximately one line of text and that text included at least one of the following: (1) contextual details from a question (e.g., characters, actions, events, places, etc.); (2) some kind of analysis using themes or elements from the questions (e.g., "that ghosts suffer from hunger and desire. I believe that all spirits do exist: It's my personal belief"); or (3) a description of how they made response decisions (e.g., "Think = fact, while believe = unverifiable truths that those still believe to hold self evident"). People failed if they did not meet the above conditions. Finally, participants answered demographics questions and were given a small thank-you gift for their time.

Primary analyses and results. Our preregistered analyses described below confirmed our hypotheses (see next section), but they were not the most stringent or sophisticated model of our data: Among other things, they did not fully exploit our repeated-measures design, because they used proportion of trials as the dependent variable; and they did not take into account the possibility that individual items may have elicited stronger or weaker responses from participants. Thus, for all studies in this paper, we focused our interpretations on series of mixed effects logistic regressions, which were the primary analyses reported in the main text.

We first conducted a mixed effects logistic regression predicting how likely a participant was to choose “believe” (or its counterparts in other languages) for a given trial, based on the superordinate category of the question (religious vs. matter-of-fact items), field site (US, Ghana, Thailand, China, or Vanuatu), and an interaction between them, with a maximal random effects structure (random intercepts and slopes by subject, and random intercepts by item). As reported in the main text, this analysis confirmed that participants were generally more likely to select “believe” to complete religious vs. matter-of-fact sentences, as predicted, even controlling for differences across sites in the overall usage of “believe” and its counterparts. It further revealed that this distinction was more pronounced in our samples from the US and Thailand, less pronounced in our sample from Ghana, and did not differ from the grand mean in our samples from China or Vanuatu. The full results of this model are in Table S3. See Fig. 1 for a visualization of responses by item category, and Fig. S1 for a visualization of responses to individual items, by sub-category.

We then conducted identical mixed effects logistic regressions for each field site individually, using the most maximal random effects structure feasible across samples (i.e., that did not cause convergence problems or perfectly correlated random effects in any sample). As reported in the main text, these analyses confirmed that the predicted effect was present in each field site considered alone; see Table S4.

In addition to the analyses reported in the main text, we also conducted a mixed effects logistic regression treating field site as a random, rather than fixed, effect. Again, the predicted effect was significant; see Table S5.

Finally, we conducted a mixed effects logistic regression parallel to that presented in Table S3 accounting for five sub-categories of items (rather taking into account only the superordinate two-way distinction between “religious” vs. “matter-of-fact” items); see Table S6. Again, the predicted effect—the difference between the two sub-categories of religious items and the three sub-categories of matter-of-fact items—was significant (contrast A). More interestingly, this analysis supported our interpretation of this finding by failing to support two of the “alternative explanations” we considered in the main text: contrary to an account that would suggest that participants used “believe” merely to mark uncertainty, participants were *not* more likely to use “believe” (or counterparts) for less widely known items than for more widely known items (contrast D); contrary to an account that would suggest that participants used “believe” merely to indicate their own disagreement with the content, participants were actually more likely to use “believe” (or counterparts) for religious items from the locally salient religion (contrast B), particularly in the two most devoutly Christian sites, Ghana and Vanuatu (see also Fig. S1). The US results clearly replicated those reported in Heiphetz, Landers, and Van Leeuwen’s (2021) Study 2.

Preregistered analyses and results. The preregistered analysis for Study 1 included a 5 (field site: US, Ghana, Thailand, China, Vanuatu) x 2 (item category: religious vs. matter-of-fact) mixed ANOVA with repeated measures on the second factor and the proportion of trials on which participants circled “believe” (or counterparts) as the dependent measure. As predicted, the main effect of item category was significant, confirming that participants tended to circle “believe” (or counterparts) for a much greater proportion of religious items (80%) than they did for matter-of-fact items (39%). The main effects of field site and the interaction between item category and field site were also significant; preregistered follow-up analyses indicated that participants in Ghana tended to circle the “believe” counterpart most often (67% of all trials) and participants in China least often (54%); and that the difference between religious and matter-of-

fact items was significant in each field site considered alone, though most pronounced in Thailand and least pronounced in Ghana. See Table S7 for the ANOVA, and Table S8 for summary statistics and follow-up tests.

The preregistration also included an additional mixed ANOVA, identical to the previous analysis, except looking at the 5 sub-categories for items (Christian religious, Buddhist religious, widely known matter-of-fact, less widely known matter-of-fact, personal life matter-of-fact) rather than the 2 superordinate categories (religious vs. matter-of-fact). Again, both main effects and the interaction were significant. See Table S9 for the ANOVA, and Table S10 for summary statistics.

Study 2

Participants. The final sample for analysis included 388 adults (US: n=71; Ghana: n=46; Thailand: n=98; China: n=100; Vanuatu: n=73). See Table S1 for demographic information. As in Study 1, we aimed to recruit approximately 50 participants per site, but retained any participants that exceeded this minimum sample size. An additional 70 participants were excluded from analyses because they completed this task after completing other experimental surveys (n=39), or because they failed the attention check (n=31).

Procedure. For all participants included in our final sample, this task was the first task included in a larger packet of survey measures. Procedure was identical to Study 1, except that instead of circling one of two words to complete each sentence, participants were asked to "complete each sentence by *writing a word in the blank*" (or they were asked for the same thing using a literal translation of this phrase in their own language). Fante-speaking participants in Ghana were asked to speak a word or phrase aloud to complete the sentence.

Coding free response data. We used a combination of hand-coding and automatic lemmatization (using the "textstem" package for R) to code whether responses included the word-stem "believe" (or counterparts), "think" (or counterparts), etc., in order to ensure that we included all possible verb tenses (e.g., "believes" and "believed") while taking into account typos and spelling variants (e.g., "belif," "bilif," and other multiple other spellings in Bislama). We considered negated phrases (e.g. "did not believe") to be instances of the target words.

Primary analyses and results. (See "Study 1" for a discussion of our primary vs. preregistered analyses.) As in Study 1, our primary analysis was a mixed effects logistic regression predicting how likely a participant was to include the word-stem "believe" (or counterparts) in their free responses to a given trial, based on the superordinate category of the question, field site, and an interaction between them, with a maximal random effects structure. This analysis confirmed that participants were generally more likely to use "believe" to complete religious vs. matter-of-fact sentences, as predicted, even controlling for differences across sites in the overall usage of "believe" and its counterparts. It further revealed that this distinction was more pronounced in our samples from the US and China, less pronounced in our samples from Ghana and Vanuatu, and did not differ from the grand mean in our sample from Thailand. The full results of this model are in Table S11. See Fig. 2 for a visualization of responses by item category, and Fig. S2 for a visualization of responses to individual items, by sub-category.

We then conducted identical mixed effects logistic regressions for each field site individually, using the most maximal random effects structure feasible across samples. As reported in the main text, these analyses confirmed that the predicted effect was present in each field site considered alone; see Table S12. The US result replicated the US findings from Heiphetz, Landers, and Van Leeuwen's (2021) Study 3.

In addition to the analyses reported in the main text, we also conducted a mixed effects logistic regression treating field site as a random, rather than fixed, effect. Again, the predicted effect was significant; see Table S13.

Finally, we conducted a mixed effects logistic regression parallel to that presented in Table S11 accounting for five sub-categories of items; see Table S14. Again, the predicted effect—the difference between the two sub-categories of religious items and the three sub-categories of matter-of-fact items—was significant (contrast A). More interestingly, this analysis supported our interpretation of this finding by failing to support two of the "alternative explanations" we considered in the main text: contrary to an account that would suggest that participants used "believe" merely to mark uncertainty, participants were *not* more likely to use "believe" (or counterparts) for less widely known items than for more widely known items (contrast D); contrary to an account that would suggest that participants used "believe" merely to indicate their own disagreement with the content, participants were actually more likely to use "believe" (or counterparts) for religious items from the locally salient religion (contrast B), particularly Vanuatu (see also Fig. S2).

Preregistered analyses and results. The preregistered analysis for Study 2 included a 5 (field site) x 2 (item category) mixed ANOVA with repeated measures on the second factor and the proportion of trials on which participants included the word-stem "believe" (or counterparts) in their free responses as the dependent measure. As predicted, the main effect of item category was significant, confirming that participants tended to use "believe" (or counterparts) for a much greater proportion of religious items (33%) than they did for matter-of-fact items (5%). The main effects of field site and the interaction between item category and field site were also significant; preregistered follow-up analyses indicated that participants in the US tended to use "believe" most often (24% of all trials) and participants in Ghana least often (8%); and that the difference between religious and matter-of-fact items was significant in each field site considered alone, though most pronounced in the US and least pronounced in Ghana. See Table S15 for the ANOVA, and Table S16 for summary statistics and follow-up tests.

The preregistration also included an additional mixed ANOVA, identical to the previous analysis, except looking at the 5 sub-categories for items (Christian religious, Buddhist religious, widely known matter-of-fact, less widely known matter-of-fact, personal life matter-of-fact) rather than the 2 superordinate categories (religious vs. matter-of-fact). Again, both main effects and the interaction were significant. See Table S17 for the ANOVA, and Table S18 for summary statistics.

Study 3

Participants. The final sample for analysis included 328 adults (US: n=57; Ghana: n=70; Thailand: n=72; China: n=49; Vanuatu: n=80). See Table S1 for demographic information. As in Studies 1-2, we aimed to recruit approximately 50 participants per site, but retained any participants that exceeded this minimum sample size. An additional 65 participants were excluded from analyses because they completed this task after completing other experimental surveys (n=49), or because they failed an attention check (n=16).

Procedure. For all participants included in our final sample, this task was the first task included in a larger packet of survey measures. Procedure was nearly identical to Study 1, except that the task consisted of 5 pairs of vignettes, all presented in the relevant language, for a total of 10 vignettes presented in one of two orders (counterbalanced across participants within each sample). In each pair of vignettes, the same key sentence appeared once in a religious context

and once in a matter-of-fact context; for example, the sentence *That's because she [thinks / believes] that aspirin is not a cure* was presented once in the context of a vignette about a member of the Church of Christ Scientist, and once in the context of a vignette about a medical student. See Table S19 for the full list of items.

Primary analyses and results. (See "Study 1" for a discussion of our primary vs. preregistered analyses.) As in Studies 1-2, our primary analysis was a mixed effects logistic regression predicting how likely a participant was to circle "believe" (or counterparts) for a given trial, based on the context in which the item was presented (religious vs. matter-of-fact vignettes), field site (US, Ghana, Thailand, China, or Vanuatu), and an interaction between them, with a maximal random effects structure (random intercepts and slopes by subject, and random intercepts by item). As reported in the main text, this analysis confirmed that participants were generally more likely to select "believe" to complete items embedded in religious vs. matter-of-fact vignettes, as predicted, even controlling for differences across sites in the overall usage of "believe" and its counterparts. It further revealed that this distinction was more pronounced in our sample from Thailand, less pronounced—to the point of absence—in our sample from Ghana, and did not differ from the grand mean in our samples from the US, China, or Vanuatu. The full results of this model are in Table S20. See Fig. 3 for a visualization of responses by vignette context, and Fig. S3 for a visualization of responses to individual items; note that these figures include an additional sample from English-speaking Ghanaian undergraduates (see "Study 3b, Ghanaian undergraduates (English)," below).

We then conducted identical mixed effects logistic regressions for each field site individually, using maximal random effects structures. As reported in the main text, these analyses confirmed that the predicted effect was present in the samples from the US, Thailand, China, and Vanuatu, but not in the sample from Ghana; see Table S21.

In addition to the analyses reported in the main text, we also conducted a mixed effects logistic regression treating field site as a random, rather than fixed, effect. Again, the predicted effect was significant; see Table S22. The US results clearly replicated Heiphetz, Landers, and Van Leeuwen's (2021) Study 4.

Preregistered analyses and results. The preregistered analysis for Study 3 consisted of a 5 (field site) x 2 (context) mixed ANOVA with repeated measures on the second factor and the proportion of trials on which participants chose "believe" (or counterparts) as the dependent measure. As predicted, the main effect of item category was significant, confirming that participants tended to choose "believe" (or counterparts) for a much greater proportion of religious items (67%) than they did for matter-of-fact items (47%). The main effects of field site was not significant, but the interaction between item category and field site was significant; preregistered follow-up analyses indicated that the difference between religious and matter-of-fact vignettes was significant in samples from the US, Thailand, China, and Vanuatu considered alone, though most pronounced in Thailand, and not significant in Ghana. See Table S23 for the ANOVA, and Table S24 for summary statistics and follow-up tests.

<u>Study 3b, Ghanaian undergraduates (English)</u>

The differences in Studies 1-3 between our results from Ghana vs. our other four sites—in particular, the attenuation (or absence) of the primary predicted effect in Ghanaian samples—suggested an intruiguing possibility: Is it more common in Ghana than in other locations for people to have a matter-of-fact attitude toward supernatural contents? The possibility of such a

combination—a matter-of-fact belief that has supernatural content—has been discussed at a theoretical level (Van Leeuwen, 2014), but it has never been documented empirically.

At the same time, there were substantial differences between our samples and methods which might have accounted for the observed differences between Ghana and other sites. In Ghana, participants were recruited in a rural location, and samples included participants from a wider range of ages who were generally from a lower socioeconomic status and had much less formal education relative to other samples (who were all young adult undergraduates attending universities in urban areas). In addition, as described above, these studies were conducted orally in Ghana, in contrast to the pen-and-paper survey administered in other sites.

In order to explore the possibility that it may be more common in Ghana for people to espouse a matter-of-fact belief about supernatural content, we recruited a sample of Ghanaian participants whom we judged would be *least* likely to have such attidues, i.e., *most* likely to resemble participants at other sites in terms of distinguishing matter-of-fact and religious belief: undergraduates attending an urban university where the language of instruction is English. Our logic was that if this group of participants also showed strong differences from particpants in other locations, that would be good evidence for a pervasive cultural difference in Ghana vs. other sites.

Participants. This additional sample included 50 Ghanaian, English-speaking undergraduates. See Table S1 for demographic information. All participants passed the attention check; this was the only task administered to this sample at this time.

Procedure. Participants completed the task described under Study 3, above. The instructions and items were in English and particpants responded using pen and paper.

Analyses and results. Adding this sample to our primary multi-site analyses did not change any of the conclusions described above. In a mixed effects logistic regression, the predicted effect fell just short of our cutoff for significance; see Table S21. In a paired t-test, it was significant; see Table S24.

Discussion. English-speaking Ghanaian undergraduates demonstrated a weak tendency to distinguish religious from matter-of-fact attitude ascriptions on the basis of vignette context—but even among these highly-educated, English speakers, this tendency was less pronounced than in our other field sites.

Taking all of the data from Ghana together, it is clear that participants in Ghana, like participants in other sites, recognized differences in attitude type as a dimension of variation in mental states, and used different words to express these differences. However, the specific correspondence between religious contents and contexts, on the one hand, and the hypothesized "believe" counterpart (*gye dzi*), on the other, was more complicated in Ghana.

Ethnographers have often commented on the centrality of talk about the supernatural in Ghana (Dulin, 2020; Dzkoto, 2020). This may provide a cultural invitation for people to have matter-of-fact beliefs with supernatural contents, as discussed above—to think about Jesus, for example, in the same a matter-of-fact way that they might think about rain. Such talk might also invite people to take the attitude of religious belief toward a topic that would appear non-religious to an outsiders—to believe that it will rain in the same way that they might believe that Jesus will return. In combination, these tendencies would explain the observed attenuation of the main predicted effect in our Ghanaian samples. We consider this a fruitful area of future research.

Extended acknowledgements

Special thanks to Larisa Heiphetz for her help conceptualizing and preregistering these studies. Thanks also to Dan Weiskopf for early discussions that led to our hypotheses and to Kilu von Prince for extensive discussion of an earlier draft of this paper. Thanks also to Jonathan Jong for discussion of an earlier draft and to Rebecca Tuvel for feedback while the present manuscript was undergoing revision. We are also grateful to three anonymous referees for *Open Mind* and to the journal editors for helpful feedback.

The larger Mind and Spirit Project involved large research teams in each of these five field sites, without whom this work would have been impossible.

The US team was led by Nikki Ross-Zehnder and Josh Brahinsky, and included Lucy Moctezuma, Chen Tan, Dominic Locantore, and Viet-Co Tran. Moctezuma, Tan and Locantore were responsible for most of the data entry for all sites. Ross-Zehnder oversaw all data management.

The Ghana team was led by John C. Dulin and Vivian A. Dzokoto. The team would especially like to thank Eunice Otoo, Francis Ekow Quartey, Kojo, and Edwina Kwakye-Gyamfi for data collection on this study.

The Thailand team was led by Felicity Aulino. The team would especially like to thank Cheewintha Boon-Long and Sangwan Palee for data collection on this study, and Pattaraporn Tripiyaratana for translation.

The China team was led by Emily Ng. The team would especially like to thank Yifan Yang for data collection on this study.

The Vanuatu team was led by Rachel E. Smith. The team would especially like to thank Jill Hinge and Polinda Lango for data collection on this study.

The Mind and Spirit Project also benefitted from the administrative support of Emily Bishop and Jen Kidwell as well as the wisdom of a group of advisors who convened annually for three years to provide feedback on the project, including (in alphabetical order) Rita Astuti, Jon Bialecki, Julia Cassaniti, Joanna Cook, Martin Fortier-Davy, Suzanne Gaskins, Nicholas Gibson, Alison Gopnik, Courtney Handman, Paul Harris, Adisorn Juntrasook, Lianne Kurina, Cristine Legare, Hazel Markus, Francesca Mezzenzana, Padmavati Ramachandran, Joel Robbins, Andrew Shtulman, Ann Taves, Hema Tharoor, Michiel van Elk, Tom Weisner, Henry Wellman, and Ciara Wirth.

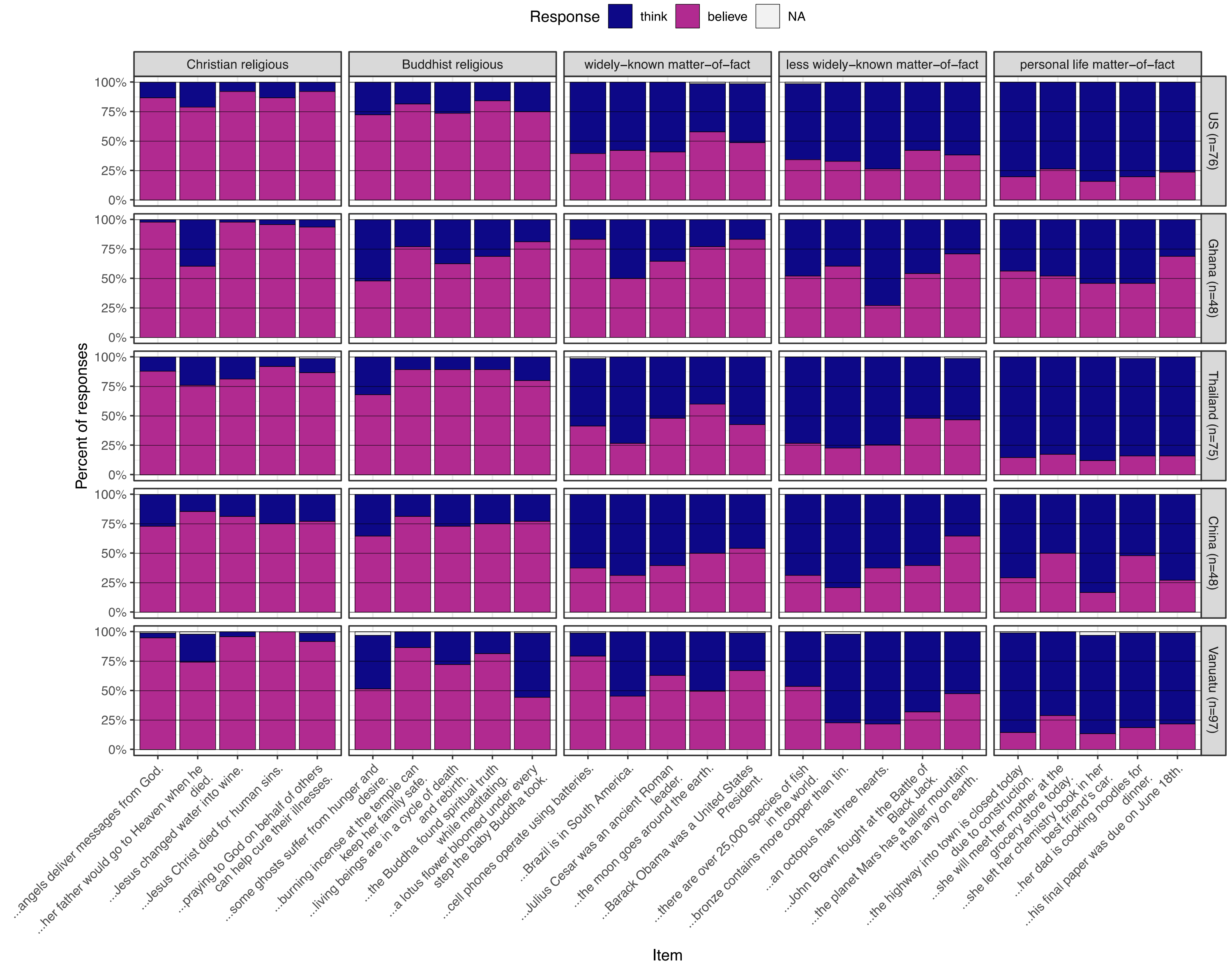

Fig. S1. Study 1: Responses to individual items, by item sub-category and field site.

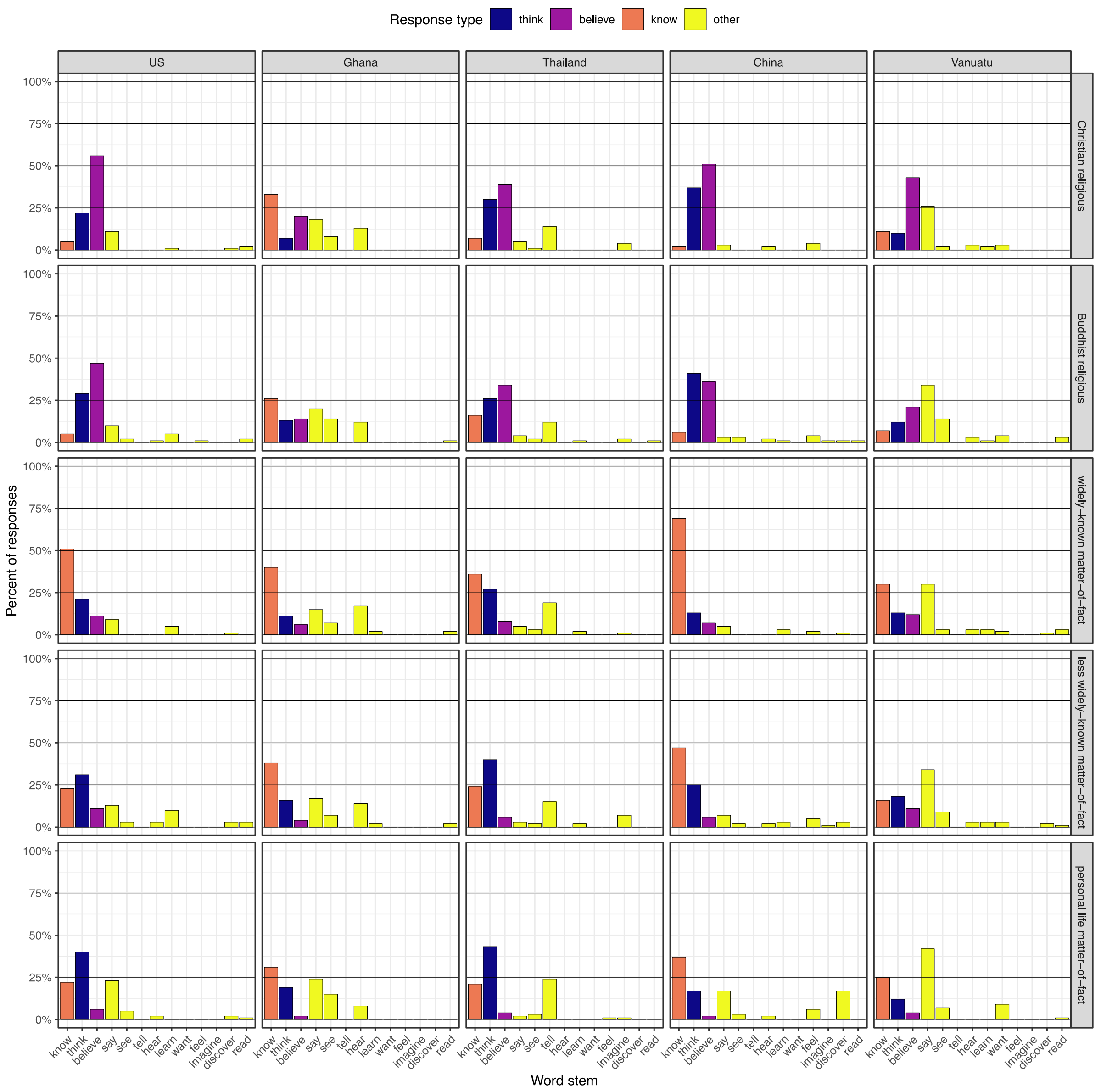

Fig. S2. Study 2: Responses to individual items, by item sub-category and field site.

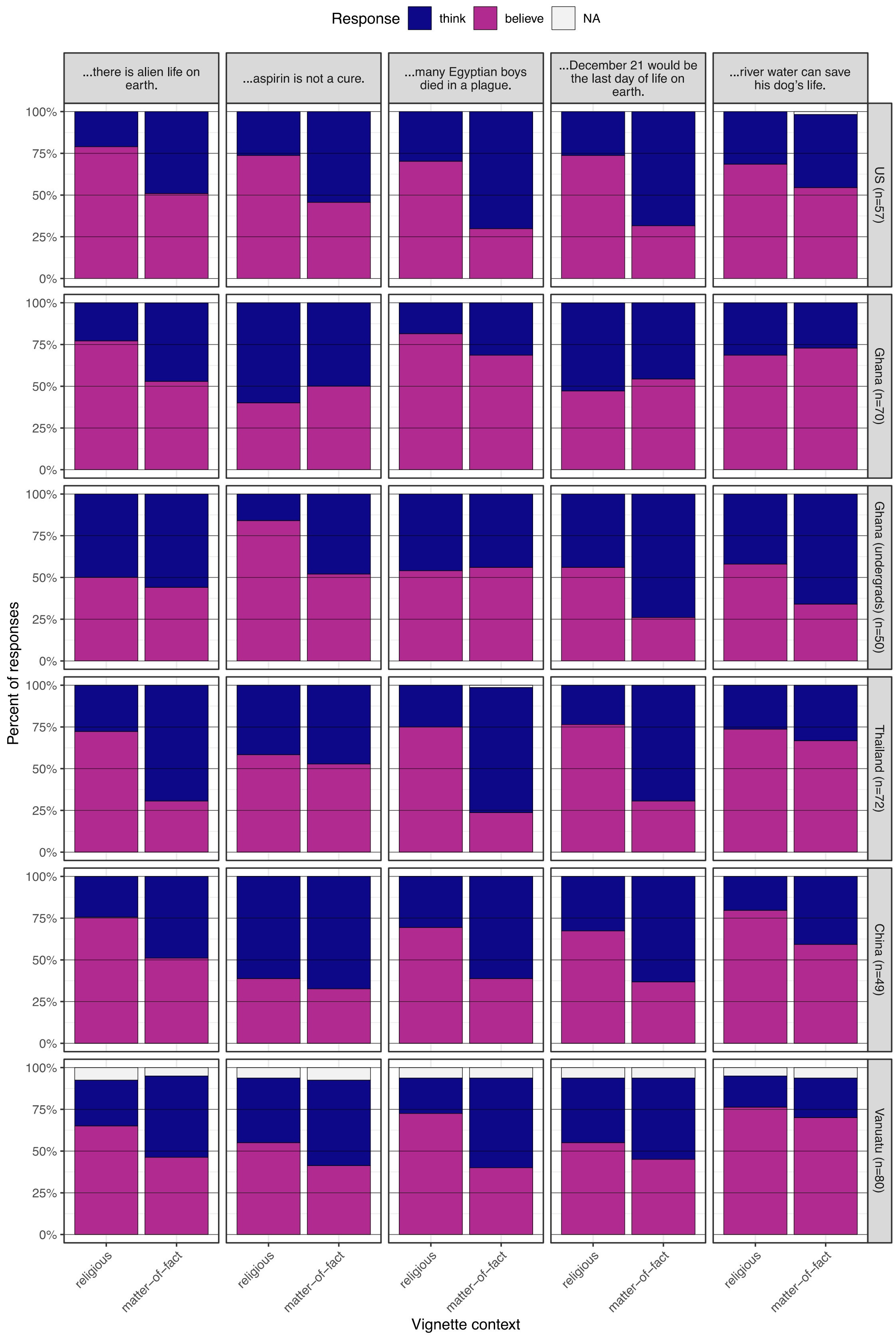

Fig. S3. Study 3: Responses to individual items, by vignette context and field site.

Table S1.

Demographic information for participants in Studies 1-3.

			Age (y)		Gender[1]			Religiosity[3]								Religious affiliation[4]				
	**Site**	**n**	**Range**	**Mean**	**m**	**f**	**Eng.**[2]	**(a)**	**(b)**	**(c)**	**(d)**	**(e)**	**(f)**	**(g)**	**(h)**	**Chr.**	**Bud.**	**Oth.**	**None**	**NA**
S1	US	76	18-29	19.84	42%	53%	93%	2.19	2.29	1.53	1.59	0.63	0.61	2.39	2.73	7%	–	5%	83%	5%
	Gh.	48	13-60	29.60	62%	38%	42%	4.83	4.79	3.38	2.83	0.58	0.67	2.65	4.12	79%	–	8%	12%	–
	Th.	75	18-23	19.37	25%	69%	32%	–	–	2.51	1.87	2.16	1.95	2.56	3.52	12%	17%	8%	61%	1%
	Ch.	48	18-22	19.85	44%	56%	94%	1.31	1.27	0.69	0.81	0.85	0.64	2.04	2.92	4%	–	–	96%	–
	Va.	97	16-29	20.26	30%	62%	73%	3.90	4.26	3.58	2.86	0.30	0.42	2.30	2.55	54%	–	6%	39%	1%
S2	US	71	18-30	19.55	58%	39%	94%	2.23	2.18	1.65	1.59	0.71	0.41	2.13	2.43	8%	1%	4%	86%	–
	Gh.	46	18-65	31.11	39%	61%	46%	4.89	4.87	3.54	2.98	0.57	0.61	3.04	4.30	87%	–	11%	2%	–
	Th.	98	18-22	19.45	78%	20%	34%	–	–	2.29	1.59	2.21	1.82	2.36	3.64	4%	17%	9%	64%	5%
	Ch.	100	18-24	19.85	49%	50%	98%	1.15	1.25	0.53	0.78	0.72	0.61	1.96	2.54	2%	1%	3%	80%	3%
	Va.	73	16-25	20.04	45%	42%	67%	3.76	3.79	3.50	2.88	0.39	0.56	2.01	2.58	37%	–	9%	51%	3%
S3	US	57	18-26	19.53	47%	51%	98%	2.21	2.11	1.50	1.63	0.46	0.45	2.40	1.98	9%	–	2%	86%	4%
	Gh.	70	18-70	29.69	44%	56%	49%	4.46	4.60	3.17	2.93	0.16	0.31	3.03	3.59	96%	–	1%	3%	–
	Gh.	50	18-28	20.92	46%	54%	58%	4.88	5.00	3.60	2.58	0.92	0.98	2.24	4.18	76%	–	20%	2%	2%
	Th.	72	18-21	18.89	28%	72%	36%	–	–	2.61	1.68	2.09	1.85	2.65	3.43	15%	18%	7%	58%	1%
	Ch.	49	18-23	19.81	51%	49%	96%	1.27	1.25	0.54	0.78	0.90	0.88	1.92	2.20	4%	–	4%	80%	2%
	Va.	80	15-29	19.13	30%	58%	49%	3.41	3.94	3.14	2.67	0.49	0.57	2.21	2.28	36%	–	4%	56%	4%

Notes:

1. *“Male” and “female” were not exhaustive categories for gender/sex. Note that this question was framed as “gender” for participants.*
2. *Participants answered 8 questions designed to gauge the degree and nature of their religiosity:*
    a. *How religious are you? (0 = not religious at all, 6 = extremely religious) [not asked in Thailand]*
    b. *How important to you is your religious practice? (0 = not important at all, 6 = of utmost importance) [not asked in Thailand]*
    c. *How often do you attend places of worship? (0 = never, 1 = once a year or less, 2 = a few times a year, 3 = once or twice a month, 4 = every week or more often)*
    d. *What best describes your level of belief in God? (0 = not believe at all, 1 = believe slightly, 2 = believe moderately, 3 = believe strongly)*
    e. *What best describes your level of belief in Buddha? (same response scale as [d])*
    f. *What best describes your level of belief in another supernatural being (other than God or Buddha)? (same response scale as [d])*
    g. *What best describes your attitude toward the supernatural? (0 = There is no such thing as supernatural forces or beings, 1 = We cannot know if there are supernatural forces and beings, 2 = There might be supernatural forces and beings, 3 = Supernatural forces and beings exist but we cannot know what they are like, 4 = There definitely are supernatural forces and beings)*

h. How important to you is your attitude toward the supernatural? (0 = not important at all, 6 = of utmost importance)

3. *Participants were asked two questions about language exposure: "What is your first language (mother tongue)?" and "Do you speak any other languages?" For the purposes of this table, if they mentioned English in either of these questions, we considered them to have been exposed to English. However, we note that participants who were completing the task in English (US participants in all studies, and Ghanaian undergraduates in Study 3), may have neglected to explicitly name English in these questions because it was implied by their completing the (written) study in English.*
4. *We coded free responses to the question "Are you part of any religious group? If yes, please list" as Christian ("Chr."), Buddhist ("Bud."), or no religion ("No"). Responses that did not clearly correspond to one of these four categories (e.g., "Muslim"; "spiritual but not religious") were coded as "Other," as were responses of "yes" with no further information about specific affiliations.* ***In retrospect, we do <u>not</u> consider this a particularly good index of religious affiliation, especially in Thailand and Vanuatu****; from our other work with very similar samples, we would strongly suspect that the vast majority of participants in both Ghana and Vanuatu were practicing Christians, and the vast majority of participants in Thailand were in some sense practicing Buddhists (though likely not affiliated with a particular, stable "Buddhist" group).*

Table S2.
English text of items included in Studies 1-2. In Study 1, participants were instructed to choose one of the words included in brackets. In Study 2, the bracketed phrase was replaced with a blank space for participants to write in their responses.

Order A	B	Item text	Category	Subcategory
1	11	*Sheila [thinks / believes] that some ghosts suffer from hunger and desire.*	religious	Buddhist
2	12	*Molly and Steven [think / believe] that the highway into town is closed today due to construction.*	matter-of-fact	personal life
3	13	*Julian [believes / thinks] that angels deliver messages from God.*	religious	Christian
4	14	*Sharon [thinks / believes] that she will meet her mother at the grocery store today.*	matter-of-fact	personal life
5	15	*Barbara used to [think / believe] that her father would go to Heaven when he died.*	religious	Christian
6	16	*Astrid [thinks / believes] that there are over 25,000 species of fish in the world.*	matter-of-fact	less known
7	17	*Nick [believes / thinks] that bronze contains more copper than tin.*	matter-of-fact	less known
8	18	*Greg and Theodore [believe / think] that cell phones operate using batteries.*	matter-of-fact	widely known
9	19	*Audrey [thinks / believes] that she left her chemistry book in her best friend's car.*	matter-of-fact	personal life
10	20	*Patrick [thinks / believes] that Brazil is in South America.*	matter-of-fact	widely known
11	21	*Lisa [thinks / believes] that burning incense at the temple can keep her family safe.*	religious	Buddhist
12	22	*Zane [believes / thinks] that Jesus changed water into wine.*	religious	Christian
13	23	*Fred and Yuriana [think / believe] that Julius Cesar was an ancient Roman leader.*	matter-of-fact	widely known
14	24	*Sarah declined going to dinner with her friend, [believing / thinking] that her dad is cooking noodles for dinner.*	matter-of-fact	personal life
15	25	*Harry [thinks / believes] that Jesus Christ died for human sins.*	religious	Christian
16	1	*Gustav [thinks / believes] that his final paper was due on June 18th.*	matter-of-fact	personal life
17	2	*Sam used to [think / believe] that living beings are in a cycle of death and rebirth.*	religious	Buddhist

18	3	*Quentin [believes / thinks] that the moon goes around the earth.*	matter-of-fact	widely known
19	4	*Penelope [believes / thinks] that an octopus has three hearts.*	matter-of-fact	less known
20	5	*Sarah and Frank [think / believe] that John Brown fought at the Battle of Black Jack.*	matter-of-fact	less known
21	6	*Katie [believes / thinks] that the planet Mars has a taller mountain than any on earth.*	matter-of-fact	less known
22	7	*Dwayne [thinks / believes] that the Buddha found spiritual truth while meditating.*	religious	Buddhist
23	8	*Sarah [believes / thinks] that praying to God on behalf of others can help cure their illnesses.*	religious	Christian
24	9	*Joe [thinks / believes] that a lotus flower bloomed under every step the baby Buddha took.*	religious	Buddhist
25	10	*Jill and Billy [think / believe] that Barack Obama was a United States President.*	matter-of-fact	widely known

Table S3.

Study 1, primary analysis: a mixed effects logistic regression predicting how likely a participant was to choose "believe" (or its counterpart in other languages) based on the superordinate category of the question ("religious" vs. "matter-of-fact" items), field site (US, Ghana, Thailand, China, or Vanuatu), and an interaction between them, with a maximal random effects structure (random intercepts and slopes by subject, and random intercepts by item). All categorical items were effect-coded for comparison to the grand mean (GM). The parameter of primary interest is in bold.

Parameter	β	Std. Err.	df	t	p	
Intercept	0.60	0.03	24.00	23.59	<0.001	***
**Category (religious vs. GM)**	**0.20**	**0.03**	**26.58**	**7.61**	**<0.001**	***
Field site (Ghana vs. GM)	0.09	0.01	338.99	6.71	<0.001	***
Field site (Thailand vs. GM)	-0.03	0.01	338.99	-2.44	0.015	*
Field site (China vs. GM)	-0.03	0.01	338.99	-2.14	0.033	*
Field site (Vanuatu vs. GM)	-0.01	0.01	338.99	-1.23	0.221	
Category (religious) × Field site (Gh.)	-0.10	0.02	339.01	-5.67	<0.001	***
Category (religious) × Field site (Th.)	0.07	0.02	339.01	4.27	<0.001	***
Category (religious) × Field site (Ch.)	-0.01	0.02	339.01	-0.55	0.585	
Category (religious) × Field site (Va.)	0.00	0.01	339.01	0.33	0.744	
"Field site" recoded (dropping Va. instead of US):						
Field site (US vs. GM)	-0.02	0.01	339.00	-1.86	0.064	
Category (religious) × Field site (US)	0.04	0.02	338.99	2.81	0.005	**

Random effects:

Group	Type	Variance	Std. Dev.
Item	Intercept	0.01	0.12
Individual	Intercept	0.00	0.06
Individual	Slope (Category)	0.01	0.12
Residual		0.17	0.41

Table S4.

Study 1, secondary analysis: mixed effects logistic regressions for each sample considered alone, predicting how likely a participant was to choose "believe" (or counterparts) based on the superordinate category of the question ("religious" vs. "matter-of-fact" items, with the most maximal random effects structure we were able to fit for all samples (random intercepts by subject and item, with no random slopes). In the interest of space, only fixed effects are reported here. All categorical items were effect-coded for comparison to the grand mean (GM). In each case, the parameter of primary interest is in bold.

Country	Parameter	β	Std. Err.	df	t	p	
US	Intercept	0.58	0.02	26.45	26.45	<0.001	***
	**Category (religious)**	**0.24**	**0.02**	**23.00**	**11.51**	**<0.001**	***
Ghana	Intercept	0.69	0.04	30.42	18.86	<0.001	***
	**Category (religious)**	**0.09**	**0.03**	**23.00**	**2.80**	**0.010**	*
Thailand	Intercept	0.57	0.03	29.64	20.37	<0.001	***
	**Category (religious)**	**0.27**	**0.03**	**23.00**	**10.08**	**<0.001**	***
China	Intercept	0.57	0.03	31.80	22.90	<0.001	***
	**Category (religious)**	**0.19**	**0.02**	**23.00**	**8.55**	**<0.001**	***
Vanuatu	Intercept	0.59	0.04	23.38	14.26	<0.001	***
	**Category (religious)**	**0.20**	**0.04**	**23.00**	**4.95**	**<0.001**	***

Table S5.

Study 1, supplemental analysis: a mixed effects logistic regression similar to that presented in Table S3, treating field site as a random rather than fixed effect, with a maximal random effects structure (random intercepts and slopes by subject, nested within country, and random intercepts by item). All categorical items were effect-coded for comparison to the grand mean (GM). The parameter of primary interest is in bold.

Parameter	β	Std. Err.	df	t	p	
Intercept	0.60	0.03	16.03	18.40	<0.001	***
**Category (religious vs. GM)**	**0.20**	**0.04**	**10.32**	**5.17**	**<0.001**	***

Random effects:

Group	Type	Variance	Std. Dev.
Country	Intercept	0.00	0.05
Country	Slope (Category)	0.00	0.06
question	Intercept	0.01	0.12
Individual, nested within Country	Intercept	0.00	0.06
Individual, nested within Country	Slope (Category)	0.01	0.12
Residual		0.17	0.41

Table S6.

Study 1, supplemental analysis: a mixed effects logistic regression similar to Table S3, examining 5 sub-categories of items. Sub-category was coded with the following planned orthogonal contrasts: (A) Religious vs. matter-of-fact items; (B) Locally salient vs. other religious items, where the "locally salient" religious was Christianity in the US, Ghana, and Vanuatu, and Buddhism in Thailand and China; (C) Widely and less widely known vs. personal life matter-of-fact items; and (D) widely vs. less widely known matter-of-fact items.

Parameter	β	Std. Err.	df	t	p	
Intercept	0.56	0.02	22.76	34.26	<0.001	***
Contrast A	0.08	0.01	20.27	12.25	<0.001	***
Contrast B	0.05	0.01	197.72	5.61	<0.001	***
Contrast C	0.06	0.01	20.27	3.97	<0.001	***
Contrast D	0.07	0.03	20.27	2.73	0.013	*
Country (Ghana vs. GM)	0.11	0.01	339.00	7.88	<0.001	***
Country (Thailand vs. GM)	-0.04	0.01	339.00	-3.46	<0.001	***
Country (China vs. GM)	-0.03	0.01	339.00	-1.88	0.061	
Country (Vanuatu vs. GM)	-0.01	0.01	339.00	-1.25	0.213	
Contrast A × Country (Gh.)	-0.04	0.00	8216.00	-9.65	<0.001	***
Contrast B × Country (Gh.)	0.06	0.03	52.18	2.29	0.026	*
Contrast C × Country (Gh.)	-0.03	0.01	8216.00	-3.02	0.003	**
Contrast D × Country (Gh.)	0.02	0.02	8216.00	1.49	0.137	
Contrast A × Country (Th.)	0.03	0.00	8216.00	7.26	<0.001	***
Contrast B × Country (Th.)	-0.06	0.03	25.91	-1.81	0.082	
Contrast C × Country (Th.)	0.02	0.01	8216.00	2.56	0.010	*
Contrast D × Country (Th.)	-0.02	0.01	8216.00	-1.37	0.170	
Contrast A × Country (Ch.)	0.00	0.00	8216.00	-0.93	0.352	
Contrast B × Country (Ch.)	-0.07	0.03	30.13	-2.12	0.042	*
Contrast C × Country (Ch.)	-0.04	0.01	8216.00	-3.73	<0.001	***
Contrast D × Country (Ch.)	-0.05	0.02	8216.00	-2.98	0.003	**
Contrast A × Country (Va.)	0.00	0.00	8216.00	0.56	0.579	
Contrast B × Country (Va.)	0.07	0.02	35.57	3.05	0.004	**
Contrast C × Country (Va.)	0.04	0.01	8216.00	5.10	<0.001	***
Contrast D × Country (Va.)	0.06	0.01	8216.00	4.48	<0.001	***
"Field site" recoded (dropping Va. instead of US):						
Field site (US vs. GM)	-0.03	0.01	339.00	-2.52	0.012	*
Contrast A × Country (US)	0.02	0.00	8216.00	4.79	<0.001	***
Contrast B × Country (US)	0.00	0.02	39.75	0.00	0.999	
Contrast C × Country (US)	0.01	0.01	8216.00	0.78	0.437	
Contrast D × Country (US)	-0.01	0.01	8216.00	-0.96	0.338	

Random effects:

Group	Type	Variance	Std. Dev.
Item	Intercept	0.01	0.08
Individual	Intercept	0.07	0.06
Residual		0.18	0.42

Table S7.

Study 1, preregistered analysis: a 5 (field site: US, Ghana, Thailand, China, Vanuatu) x 2 (item category: religious vs. matter-of-fact) mixed ANOVA with repeated measures on the second factor and the proportion of trials on which participants chose "believe" (or counterparts) as the dependent measure. The effect of primary interest is in bold.

Effect	df (numerator)	df (denominator)	F	p		η^2_G
Country	4	339	11.39	<0.001	***	0.039
**Category**	**1**	**339**	**580.44**	**<0.001**	***	**0.502**
Country × Category	4	339	11.22	<0.001	***	0.078

Table S8.

Study 1, summary statistics for the mean proportion of responses on which a participant chose "believe" (or counterparts), by field site and item category, as well as follow-up analyses to the ANOVA presented in Table S7. T-values, degrees of freedom, and p-values are from paired t-tests comparing the proportion of "believe" responses by category for individual participants.

Field site	All items	Religious items	Matter-of-fact items	Mean diff. [95% CI]	t	df	p	
US	0.53	0.82	0.34	0.49 [0.40, 0.57]	11.18	75	<0.001	***
Ghana	0.67	0.78	0.59	0.19 [0.13, 0.25]	6.64	47	<0.001	***
Thailand	0.52	0.84	0.31	0.53 [0.47, 0.59]	16.59	74	<0.001	***
China	0.54	0.76	0.38	0.38 [0.27, 0.48]	7.38	47	<0.001	***
Vanuatu	0.55	0.79	0.39	0.41 [0.36, 0.45]	17.05	96	<0.001	***
Overall	0.55	0.80	0.39	0.42 [0.38, 0.45]	24.78	343	<0.001	***

Table S9.

Study 1, preregistered analysis: a 5 (field site: US, Ghana, Thailand, China, Vanuatu) x 5 (item sub-category) mixed ANOVA with repeated measures on the second factor and the proportion of trials on which participants chose "believe" (or counterparts) as the dependent measure. The effect of primary interest is in bold.

Effect	df (numerator)	df (denominator)	F	p		η^2_G
Country	4	339.00	16.10	<0.001	***	0.035
**Sub-category**	**3.14**	**1064.88**	**312.63**	**<0.001**	***	**0.393**
Country × Sub-category	12.56	1064.88	9.46	<0.001	***	0.078

Table S10.

Study 1, summary statistics for the mean proportion of responses on which a participant chose "believe" (or counterparts), by field site and item sub-category. These summary statistics are intended to shed light on the results of the ANOVA presented in Table S9.

		Religious		Matter-of-fact		
**Field site**	**All items**	**Christian**	**Buddhist**	**widely known**	**less widely known**	**personal life**
US	0.53	0.87	0.77	0.46	0.35	0.21
Ghana	0.67	0.89	0.68	0.72	0.53	0.54
Thailand	0.52	0.85	0.83	0.44	0.34	0.15
China	0.54	0.78	0.74	0.42	0.39	0.34
Vanuatu	0.55	0.91	0.67	0.61	0.35	0.19
Overall	0.55	0.87	0.74	0.53	0.38	0.26

Table S11.

Study 2, primary analysis: a mixed effects logistic regression predicting how likely a participant was to include the word "believe" (or its counterpart in other languages) in their free responses based on the superordinate category of the question ("religious" vs. "matter-of-fact" items), field site (US, Ghana, Thailand, China, or Vanuatu), and an interaction between them, with a maximal random effects structure (random intercepts and slopes by subject, and random intercepts by item). All categorical items were effect-coded for comparison to the grand mean (GM). The parameter of primary interest is in bold.

Parameter	β	Std. Err.	df	t	p	
Intercept	0.19	0.01	55.49	13.80	<0.001	***
**Category (religious vs. GM)**	0.13	0.01	40.79	10.58	<0.001	***
Field site (Ghana vs. GM)	-0.09	0.02	383.00	-4.57	<0.001	***
Field site (Thailand vs. GM)	0.01	0.02	383.00	0.55	0.584	
Field site (China vs. GM)	0.03	0.02	383.00	1.80	0.073	
Field site (Vanuatu vs. GM)	-0.03	0.02	383.00	-2.03	0.043	*
Category (religious) × Field site (Gh.)	-0.07	0.02	383.01	-4.53	<0.001	***
Category (religious) × Field site (Th.)	0.01	0.01	383.01	0.61	0.541	
Category (religious) × Field site (Ch.)	0.04	0.01	383.01	3.24	0.001	**
Category (religious) × Field site (Va.)	-0.04	0.01	383.01	-3.05	0.002	**
"Field site" recoded (dropping Va. instead of US):						
Field site (US vs. GM)	0.09	0.02	383.00	5.36	<0.001	***
Category (religious) × Field site (US)	0.07	0.01	383.01	4.99	<0.001	***

Random effects:

Group	Type	Variance	Std. Dev.
Item	Intercept	0.00	0.05
Individual	Intercept	0.02	0.15
Individual	Slope (Category)	0.01	0.12
Residual		0.08	0.29

Table S12.

Study 2, secondary analysis: mixed effects logistic regressions for each sample considered alone, predicting how likely a participant was include the word “believe” (or its counterpart in other languages) in their free responses based on the superordinate category of the question (“religious” vs. “matter-of-fact” items), with the most maximal random effects structure we were able to fit for all samples (random intercepts by subject and item, with no random slopes). In the interest of space, only fixed effects are reported here. All categorical items were effect-coded for comparison to the grand mean (GM). In each case, the parameter of primary interest is in bold.

Country	Parameter	β	Std. Err.	df	t	p	
US	Intercept	0.28	0.02	64.60	13.86	<0.001	***
	**Category (religious)**	**0.20**	**0.01**	**23.00**	**15.84**	**<0.001**	***
Ghana	Intercept	0.09	0.02	49.60	4.47	<0.001	***
	**Category (religious)**	**0.06**	**0.01**	**23.00**	**6.07**	**<0.001**	***
Thailand	Intercept	0.19	0.02	78.96	9.90	<0.001	***
	**Category (religious)**	**0.14**	**0.01**	**23.00**	**10.83**	**<0.001**	***
China	Intercept	0.21	0.02	37.51	9.44	<0.001	***
	**Category (religious)**	**0.17**	**0.02**	**23.00**	**8.64**	**<0.001**	***
Vanuatu	Intercept	0.15	0.02	62.67	7.02	<0.001	***
	**Category (religious)**	**0.09**	**0.02**	**23.00**	**5.89**	**<0.001**	***

Table S13.

Study 2, supplemental analysis: a mixed effects logistic regression similar to that presented in Table S3, treating field site as a random rather than fixed effect, with the most maximal random effects structure feasible (random intercepts by subject, nested within country, and by item). All categorical items were effect-coded for comparison to the grand mean (GM). The parameter of primary interest is in bold.

Parameter	β	Std. Err.	df	t	p	
Intercept	0.19	0.03	5.12	6.82	<0.001	***
**Category (religious vs. GM)**	**0.14**	**0.01**	**23.00**	**13.00**	**<0.001**	***

Random effects:

Group	Type	Variance	Std. Dev.
Country	Intercept	0.00	0.05
question	Intercept	0.00	0.05
Individual, nested within Country	Intercept	0.02	0.13
Residual		0.10	0.32

Table S14.

Study 2, supplemental analysis: a mixed effects logistic regression similar to Table S11, examining 5 sub-categories of items. Sub-category was coded with the following planned orthogonal contrasts: (A) Religious vs. matter-of-fact items; (B) Locally salient vs. other religious items, where the "locally salient" religious was Christianity in the US, Ghana, and Vanuatu, and Buddhism in Thailand and China; (C) Widely and less widely known vs. personal life matter-of-fact items; and (D) widely vs. less widely known matter-of-fact items.

Parameter	β	Std. Err.	df	t	p	
Intercept	0.15	0.01	53.94	14.21	<0.001	***
Contrast A	0.05	0.00	20.46	14.73	<0.001	***
Contrast B	0.01	0.01	418.34	2.31	0.022	*
Contrast C	0.01	0.01	20.46	1.73	0.098	
Contrast D	0.01	0.01	20.46	0.53	0.599	
Country (Ghana vs. GM)	-0.07	0.02	383.00	-4.09	<0.001	***
Country (Thailand vs. GM)	0.01	0.01	383.00	0.55	0.584	
Country (China vs. GM)	0.00	0.01	383.00	0.28	0.780	
Country (Vanuatu vs. GM)	-0.02	0.01	383.00	-1.48	0.139	
Contrast A × Country (Gh.)	-0.03	0.00	9272.00	-8.82	<0.001	***
Contrast B × Country (Gh.)	0.01	0.02	96.57	0.83	0.408	
Contrast C × Country (Gh.)	0.00	0.01	9272.00	-0.34	0.734	
Contrast D × Country (Gh.)	0.00	0.01	9272.00	-0.05	0.964	
Contrast A × Country (Th.)	0.00	0.00	9272.00	1.67	0.094	
Contrast B × Country (Th.)	-0.02	0.02	28.59	-1.41	0.170	
Contrast C × Country (Th.)	-0.01	0.01	9272.00	-1.09	0.274	
Contrast D × Country (Th.)	0.00	0.01	9272.00	0.34	0.733	
Contrast A × Country (Ch.)	0.01	0.00	9272.00	4.12	<0.001	***
Contrast B × Country (Ch.)	-0.08	0.02	28.37	-4.64	<0.001	***
Contrast C × Country (Ch.)	0.00	0.01	9272.00	-0.18	0.859	
Contrast D × Country (Ch.)	0.00	0.01	9272.00	0.21	0.833	
Contrast A × Country (Va.)	-0.02	0.00	9272.00	-5.73	<0.001	***
Contrast B × Country (Va.)	0.06	0.01	64.16	4.41	<0.001	***
Contrast C × Country (Va.)	0.00	0.01	9272.00	0.77	0.440	
Contrast D × Country (Va.)	0.00	0.01	9272.00	-0.02	0.982	
"Field site" recoded (dropping Va. instead of US):						
Field site (US vs. GM)	0.08	0.02	383.00	5.59	<0.001	***
Contrast A × Country (US)	0.03	0.00	9272.00	11.01	<0.001	***
Contrast B × Country (US)	0.03	0.01	65.57	2.06	0.044	
Contrast C × Country (US)	0.00	0.01	9272.00	0.77	0.441	
Contrast D × Country (US)	0.00	0.01	9272.00	-0.41	0.679	

Random effects:

Group	Type	Variance	Std. Dev.
Item	Intercept	0.00	0.04
Individual	Intercept	0.02	0.13
Residual		0.09	0.31

Table S15.

Study 2, preregistered analysis: a 5 (field site: US, Ghana, Thailand, China, Vanuatu) x 2 (item category: religious vs. matter-of-fact) mixed ANOVA with repeated measures on the second factor and the proportion of trials on which participants included the word-stem "believe" (or counterparts) in their free responses as the dependent measure. The effect of primary interest is in bold.

**Effect**	**df (numerator)**	**df (denominator)**	**F**	**p**		**η^2_G**
Country	4	383	10.87	<0.001	***	0.062
**Category**	**1**	**383**	**359.606**	**<0.001**	***	**0.246**
Country × Category	4	383	12.59	<0.001	***	0.046

Table S16.

Study 2, summary statistics for the mean proportion of responses on which a participant included the word-stem "believe" (or counterparts) in their free responses, by field site and item category, as well as follow-up analyses to the ANOVA presented in Table S15. T-values, degrees of freedom, and p-values are from paired t-tests comparing the proportion of "believe" responses by category for individual participants.

Field site	All items	Religious items	Matter-of-fact items	Mean diff. [95% CI]	t	df	p	
US	0.24	0.48	0.08	0.40 [0.33, 0.47]	11.48	70	<0.001	***
Ghana	0.08	0.15	0.03	0.11 [0.06, 0.17]	4.06	45	0.002	**
Thailand	0.17	0.33	0.06	0.28 [0.22, 0.33]	9.75	97	<0.001	***
China	0.18	0.38	0.04	0.34 [0.29, 0.40]	12.57	99	<0.001	***
Vanuatu	0.13	0.24	0.06	0.18 [0.13, 0.23]	6.83	72	<0.001	***
Overall	0.19	0.33	0.05	0.28 [0.25, 0.30]	19.79	387	<0.001	***

Table S17.

Study 2, preregistered analysis: a 5 (field site: US, Ghana, Thailand, China, Vanuatu) x 5 (item sub-category) mixed ANOVA with repeated measures on the second factor and the proportion of trials on which participants included the word-stem "believe" (or counterparts) in their free responses as the dependent measure. The effect of primary interest is in bold.

Effect	df (numerator)	df (denominator)	F	p		η^2_G
Country	4	383.00	9.92	<0.001	***	0.040
**Sub-category**	**2.29**	**876.25**	**231.17**	**<0.001**	***	**0.240**
Country × Sub-category	9.15	876.25	8.51	<0.001	***	0.046

Table S18.

Study 2, summary statistics for the mean proportion of responses on which a participant included the word-stem "believe" (or counterparts) in their free responses, by field site and item sub-category. These summary statistics are intended to shed light on the results of the ANOVA presented in Table S17.

		**Religious**			**Matter-of-fact**		
**Field site**	**All items**	**Christian**	**Buddhist**		**widely known**	**less widely known**	**personal life**
US	0.24	0.52	0.44	0.10	0.09	0.04	0.24
Ghana	0.08	0.17	0.12	0.05	0.04	0.01	0.08
Thailand	0.17	0.35	0.32	0.07	0.06	0.04	0.17
China	0.18	0.45	0.32	0.07	0.04	0.02	0.18
Vanuatu	0.13	0.32	0.16	0.08	0.07	0.02	0.13
Overall	0.17	0.38	0.29	0.08	0.06	0.03	0.17

Table S19.

English text of items included in Studies 3. As in Study 1, participants were instructed to choose one of the two words included in brackets.

Order A	B	Item text	Pair	Context
1	6	*Max is a member of the research organization called NASA. He has studied astronomy for many years. He always reads NASA's research reports. Today he read in a research report that a rock from outer space crashed on earth, carrying alien bacteria. Max now [thinks / believes] that there is alien life on earth.*	A	matter-of-fact
2	7	*Kerry had bad headaches in the afternoons all last year. Sometimes her friends offered her aspirin. But Kerry took courses at a medical school. That school teaches that drinking water is the way to cure a headache and aspirin is not. So Kerry always refused the aspirin her friends offered. That's because she [thinks / believes] that aspirin is not a cure.*	B	matter-of-fact
3	8	*Jeff is a member of the church of Scientology. He has practiced that religion for many years. He always reads the church's sacred texts. Today he read in his holy book that powerful alien beings from outer space long ago came to live on earth. Jeff now [believes / thinks] that there is alien life on earth.*	A	religious
4	9	*Stuart goes to church every Sunday, and he reads the Bible every day. His preacher says that God punished the Egyptians for not freeing the Israelites from slavery. According to the preacher, God sent ten plagues to the Egyptians, including the death of every firstborn son. Stuart [thinks / believes] that many Egyptian boys died in a plague.*	C	religious
5	10	*Sim's dog is very sick. The dog drank dirty water from the well. So Sim visits a holy man in his community who talks to the gods. That man says that a river to the West has special magical water. Sim sets off with his dog to find the river. Sim [believes / thinks] that river water can save his dog's life.*	D	religious
6	1	*The ancient religion from the Mayan civilization had a calendar. That calendar said the world would end when the calendar ends. Jim was a devout follower of this Mayan religion. He used to perform sacrifices to Mayan gods. In 2012, Jim figured out that the Mayan calendar says the world ends on December 21, 2012. At that time, Jim [thought / believed] that December 21 would be the last day of life on earth.*	E	religious
7	2	*Terry had bad headaches in the afternoons all last year. Sometimes her friends offered her aspirin. But Terry belonged to the Church of Christ Scientist. That church teaches that prayer is the way to cure illness and medicine is not. So Terry always refused the aspirin her friends offered. That's because she [thinks / believes] that aspirin is not a cure.*	B	religious
8	3	*Astronomers used to have a calendar of asteroids (rocks from outer space). That calendar said life would end, if a giant asteroid hit the Earth. Rick used to study astronomy carefully. He observed the sky every night. In 2012, Rick figured out from the calendar that an*	E	matter-of-fact

		enormous rock was supposed to hit earth on December 21, 2012. At that time, Rick [thought / believed] that December 21 would be the last day of life on earth.		
9	4	*Tim goes to the library every week, and he reads history every day. One history expert says that sickness in Egypt was very common when the Israelites were there. According to the history expert, many plagues happened at that time, including one deadly sickness that affected boys. Tim [thinks / believes] that many Egyptian boys died in a plague.*	C	matter-of-fact
10	5	*Ram's dog is very sick. The dog drank dirty water from the well. So Ram visits a man in his community who travels many places. That man says that a river to the West has clean healthy water. Ram sets off with his dog to find the river. Ram [believes / thinks] that river water can save his dog's life.*	D	matter-of-fact

Table S20.

Study 3, primary analysis: a mixed effects logistic regression predicting how likely a participant was to choose "believe" (or its counterpart in other languages) based on context in which the item was presented ("religious" vs. "matter-of-fact" vignettes), field site (US, Ghana, Thailand, China, or Vanuatu), and an interaction between them, with a maximal random effects structure (random intercepts and slopes by subject, and random intercepts by item). All categorical items were effect-coded for comparison to the grand mean (GM). The parameter of primary interest is in bold.

Parameter	β	Std. Err.	df	t	p	
Intercept	0.57	0.03	8.33	18.37	<0.001	***
**Context (religious vs. GM)**	**0.10**	**0.03**	**8.17**	**3.31**	**0.010**	*
Field site (Ghana vs. GM)	0.04	0.02	323.00	2.23	0.026	*
Field site (Thailand vs. GM)	-0.01	0.02	323.00	-0.75	0.454	
Field site (China vs. GM)	-0.02	0.02	323.00	-1.17	0.242	
Field site (Vanuatu vs. GM)	-0.01	0.02	323.00	-0.40	0.692	
Context (religious) × Field site (Gh.)	-0.09	0.02	323.02	-5.17	<0.001	***
Context (religious) × Field site (Th.)	0.05	0.02	323.02	2.93	0.004	**
Context (religious) × Field site (Ch.)	0.01	0.02	323.02	0.50	0.619	
Context (religious) × Field site (Va.)	-0.02	0.02	323.02	-1.33	0.183	
"Field site" recoded (dropping Va. instead of US):						
Field site (US vs. GM)	0.00	0.02	323.00	0.22	0.828	
Context (religious) × Field site (US)	0.05	0.02	323.02	2.75	0.006	**

Random effects:

Group	Type	Variance	Std. Dev.
Item	Intercept	0.01	0.09
Individual	Intercept	0.01	0.07
Individual	Slope (Context)	0.00	0.05
Residual		0.22	0.47

Table S21.

Study 3, secondary analysis: mixed effects logistic regressions for each sample considered alone, predicting how likely a participant was to choose "believe" (or counterparts) based on context in which the item was presented ("religious" vs. "matter-of-fact" vignettes), with the most maximal random effects structure we were able to fit for all samples (random intercepts by subject and item, with no random slopes). In the interest of space, only fixed effects are reported here. All categorical items were effect-coded for comparison to the grand mean (GM). In each case, the parameter of primary interest is in bold. *Note: The Ghanaian undergraduate sample described here was not included in the multi-site analyses reported in Tables S20, S22, and S23.*

Country	Parameter	β	Std. Err.	df	t	p	
US	Intercept	0.58	0.03	8.00	21.69	<0.001	***
	**Context (religious)**	**0.15**	**0.03**	**8.00**	**5.74**	**<0.001**	***
Ghana	Intercept	0.61	0.05	8.78	12.70	<0.001	***
	**Context (religious)**	**0.02**	**0.05**	**8.00**	**0.33**	**0.747**	
Gh. undergrad.	Intercept	0.51	0.04	9.40	11.95	<0.001	***
(English)	**Context (religious)**	**0.09**	**0.04**	**8.00**	**2.19**	**0.060**	
Thailand	Intercept	0.56	0.04	8.66	12.53	<0.001	***
	**Context (religious)**	**0.15**	**0.04**	**8.00**	**3.46**	**0.009**	**
China	Intercept	0.55	0.04	8.00	12.60	<0.001	***
	**Context (religious)**	**0.11**	**0.04**	**8.00**	**2.58**	**0.033**	*
Vanuatu	Intercept	0.57	0.04	10.41	15.04	<0.001	***
	**Context (religious)**	**0.08**	**0.04**	**8.00**	**2.31**	**0.049**	*

Table S22.

Study 3, secondary analysis: a mixed effects logistic regression similar to that presented in Table S20, treating field site as a random rather than fixed effect, with a maximal random effects structure (random intercepts and slopes by subject, nested within country, and random intercepts by item). All categorical items were effect-coded for comparison to the grand mean (GM). The parameter of primary interest is in bold.

Parameter	β	Std. Err.	df	t	p	
Intercept	0.56	0.03	9.73	21.67	<0.001	***
**Context (religious vs. GM)**	**0.10**	**0.03**	**11.57**	**3.24**	**0.007**	**

Random effects:

Group	Type	Variance	Std. Dev.
Country	Intercept	0.00	0.02
Country	Slope (Context)	0.00	0.05
question	Intercept	0.01	0.07
Individual, nested within Country	Intercept	0.01	0.07
Individual, nested within Country	Slope (Context)	0.00	0.04
Residual		0.22	0.47

Table S23.

Study 3, preregistered analysis: a 5 (field site: US, Ghana, Thailand, China, Vanuatu) x 2 (context: religious vs. matter-of-fact) mixed ANOVA with repeated measures on the second factor and the proportion of trials on which participants chose "believe" (or counterparts) as the dependent measure. The effect of primary interest is in bold.

Effect	df (numerator)	df (denominator)	F	p		η^2_G
Country	4	323	1.43	0.223		0.009
**Category**	**1**	**323**	**138.56**	**<0.001**	***	**0.159**
Country × Category	4	323	9.11	<0.001	***	0.050

Table S24.

Study 3, summary statistics for the mean proportion of responses on which a participant chose "believe" (or counterparts), by field site and context, as well as follow-up analyses to the ANOVA presented in Table S22. T-values, degrees of freedom, and p-values are from paired t-tests comparing the proportion of "believe" responses by category for individual participants. *Note: The Ghanaian undergraduate sample described here was not included in the multi-site analyses reported in Tables S20, S22, and S23; and was not included in the final row of this table to calculate tendencies collapsing across sites.*

Field site	All vignettes	Religious context	Matter-of-fact context	Mean diff. [95% CI]	t	df	p	
US	0.58	0.73	0.42	0.31 [0.22, 0.39]	7.50	56	<0.001	***
Ghana	0.61	0.63	0.60	0.03 [-0.03, 0.09]	1.03	69	0.308	
Gh. undergrad. (English)	0.51	0.60	0.42	0.18 [0.10, 0.26]	4.32	49	<0.001	***
Thailand	0.56	0.71	0.41	0.30 [0.23, 0.37]	8.50	71	<0.001	***
China	0.55	0.66	0.44	0.22 [0.11, 0.34]	3.88	48	<0.001	***
Vanuatu	0.57	0.65	0.48	0.16 [0.10, 0.23]	4.87	79	<0.001	***
Overall	0.57	0.67	0.47	0.20 [0.16, 0.23]	11.08	327	<0.001	***

Data S1. (separate file)
Data for Study 1 (all samples).

Data S2. (separate file)
Data for Study 2 (all samples).

Data S3. (separate file)
Data for Study 3 (all samples, including the additional sample of English-speaking Ghanaian undergraduates).

These data files, along with files for analyses and figures, are available through both OSF and GitHub.

OSF: https://osf.io/qy3js/
GitHub: https://github.com/kgweisman/think_believe
